# Descriptions of two new species of *Spartaeus* Thorell, 1891 (Araneae, Salticidae, Spartaeinae) from southern China

**DOI:** 10.3897/zookeys.1282.194782

**Published:** 2026-06-12

**Authors:** Zhenhao Luo, Yi Ni, Junxia Zhang

**Affiliations:** 1 Key Laboratory of Zoological Systematics and Application of Hebei Province, College of Life Sciences, Hebei University, Baoding, Hebei 071002, China Hebei Basic Science Center for Biotic Interaction, Hebei University Baoding China; 2 Hebei Basic Science Center for Biotic Interaction, Hebei University, Baoding, Hebei 071002, China Key Laboratory of Zoological Systematics and Application of Hebei Province, College of Life Sciences, Hebei University Baoding China; 3 Engineering Research Center of Ecological Safety and Conservation in Beijing-Tianjin-Hebei (Xiong’an New Area) of MOE, Baoding, Hebei 071002, China Engineering Research Center of Ecological Safety and Conservation in Beijing-Tianjin-Hebei (Xiong’an New Area) of MOE Baoding China

**Keywords:** Jumping spider, morphology, Spartaeina, Spartaeini, taxonomy

## Abstract

Two new species of *Spartaeus* Thorell, 1891 from southern China are described: *S. yingdeensis***sp. nov**. (♂♀) from Guangdong Province and *S. rotiscutus***sp. nov**. (♂♀) from Yunnan Province. Diagnostic illustrations and photographs are provided.

## Introduction

Within jumping spiders, family Salticidae, the subfamily Salticinae is exceptionally species-rich, accounting for over 6,000 of the nearly 7,000 known salticid species worldwide (WSC 2026). In contrast, the six non-salticine subfamilies contribute less than 10% of this overall diversity (Maddison 2015; WSC 2026). Among these, Spartaeinae is notably diverse, largely due to the tribe Spartaeini, which currently encompasses 19 genera and 134 described species (WSC 2026).

The genus *Spartaeus* Thorell, 1891, which is typically found in depressions on rock surfaces or in hollows of tree trunks (Wanless 1987), represents one of the most diverse lineages within the tribe Spartaeini. Wanless (1987) provided the first comprehensive taxonomic revision of the genus, recognizing three species and establishing diagnostic characters primarily based on genitalia structure. Yang et al. (2017) described five new species from China, significantly enhancing the known species and morphological diversity of *Spartaeus*. Recent phylogenomic analyses based on ultra-conserved elements (UCEs) have revealed a distinct lineage within Spartaeini, leading to the establishment of the genus *Protaeus* Ni, Yu & Zhang, 2025 (Zhang et al. 2024; Ni et al. 2025). The species *Spartaeus forcipiformis* Yang, Liu, Liu & Peng, 2017 was transferred to this genus based on its similarity in genitalia structures with the type species of *Protaeus* (Ni et al. 2025). Currently, *Spartaeus* comprises 19 species distributed in the Oriental region (WSC 2026), yet we anticipate a significant amount of diversity remains to be discovered.

In this study, we describe two new species of *Spartaeus* collected from karst habitats in southern China, aiming to advance our knowledge of the species diversity of this genus.

## Material and methods

Specimens preserved in 95% ethanol were examined under a Nikon SMZ 1500 stereomicroscope and measured using a Leica M205A stereomicroscope. All measurements were taken in millimetres with the dedicated measurement tool in Leica LAS v. 4.3 software. The total length was measured from the anterior median eyes to the posterior end of spinnerets; carapace length was from the anterior end of carapace to the anterior end of pedicel; abdomen length was from the posterior end of pedicel to the anterior end of anal tubercle; width was the widest part of the carapace or abdomen. Measurements of legs are shown as: total length (femur, patella, tibia, metatarsus, tarsus); leg formulas are shown as: longest to shortest. Female genitalia were cleared in a pancreatin solution (Álvarez-Padilla and Hormiga 2007). The setae of male palp were mostly removed using a Chinese acupuncture needle to ensure that its complex palpal structures were not obscured. Photographs of the ethanol-immersed specimens were captured using an Olympus BX53 microscope equipped with a Kuy Nice CCD camera. The resulting image stacks were then processed using Helicon Focus v. 8 software. Living specimens were photographed using a Nikon Z30 camera equipped with an AstrHori 120 mm F2.8 macro lens. Final images were retouched in Adobe Photoshop CC 2023. All specimens examined are deposited in the Museum of Hebei University (**MHBU**; Baoding, China).

Abbreviations used in this study: **ALE**, anterior lateral eye; **AME**, anterior median eye; **CD**, copulatory duct; **CO**, copulatory opening; **E**, embolus; **FD**, fertilization duct; **ITA**, intermediate tibial apophysis; **M**, membrane of distal haematodocha; **PLE**, posterior lateral eye; **PME**, posterior median eye; **RTA**, retrolateral tibial apophysis; **S**, spermatheca; **SM**, spermophor; **T**, tegulum; **TA**, tegular apophysis; **TD**, tegular depression; **VTA**, ventral tibial apophysis.

## Taxonomy

### Spartaeus

Taxon classificationAnimaliaAraneaeSalticidae

Thorell, 1891

91DE5C53-6D6E-5025-91FA-2CFE87737513

Spartaeus Thorell, 1891: 137.

#### Type species.

*Boethus spinimanus* Thorell, 1878, by original designation.

### Spartaeus yingdeensis sp. nov.

Taxon classificationAnimaliaAraneaeSalticidae

3B77054C-C7FB-5EB7-B0EB-4FD58DEC8809

https://zoobank.org/BC18AAD3-CDA4-402D-9B95-6791B29B659E

Figs 1, 2, 3, 4

#### Chinese common name.

英德散蛛.

#### Type material.

***Holotype***: China • ♂ (MHBU-ARA-00028588), Guangdong Province, Qingyuan City, Yingde City, Baigaiyanzhai, 24.3052°N, 113.3230°E, 107 m elev., 16.VI.2025, K. Yu, Y. Ni & T. Li leg. ***Paratypes***: China • 1♀ (MHBU-ARA-00028589), 1♂ (MHBU-ARA-00028592), Yingde City, Laiwu Village, 24.2520°N, 113.3248°E, 20 m elev., 17.VI.2025, K. Yu, Y. Ni & T. Li leg. • 1♀1J (MHBU-ARA-00028593), same data as holotype • 1♂ (MHBU-ARA-00028590), Yingde City, Makoupai, 24.2639°N, 113.3278°E, 50 m elev., 17.VI.2025, K. Yu, Y. Ni & T. Li leg. • 1♂ (MHBU-ARA-00028591), Yingde City, Boluoriver beitou, 24.3929°N, 112.9751°E, 48 m elev., 18.VI.2025, K. Yu, Y. Ni & T. Li leg.

**Figure 1.**
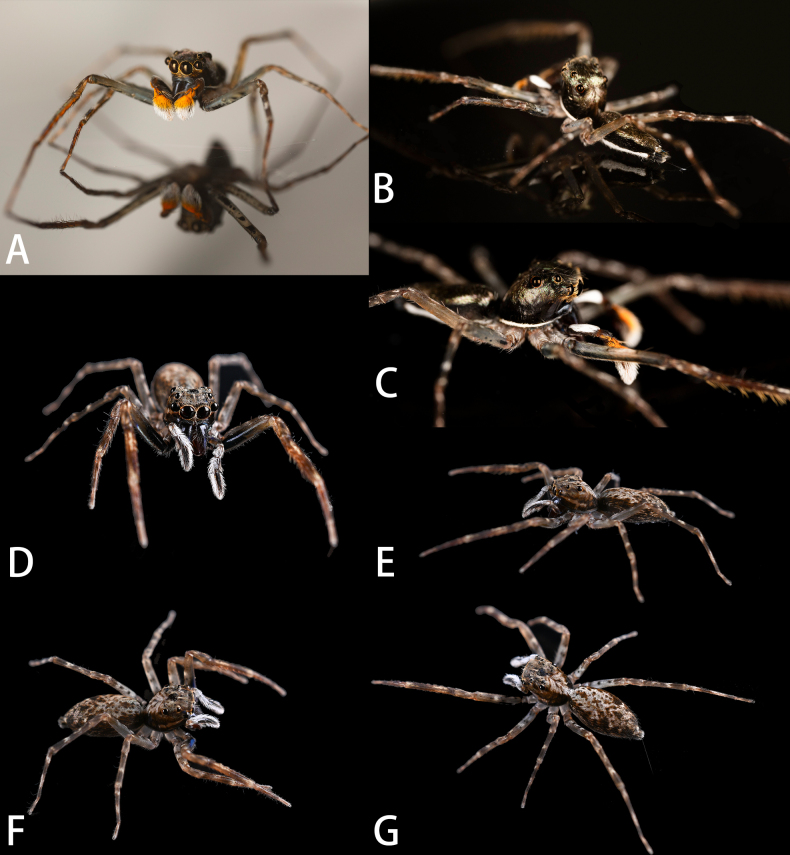
*Spartaeus yingdeensis* sp. nov., living photos. **A–C**. Holotype male; **D–G**. Paratype female (MHBU-ARA-00028589).

**Figure 2.**
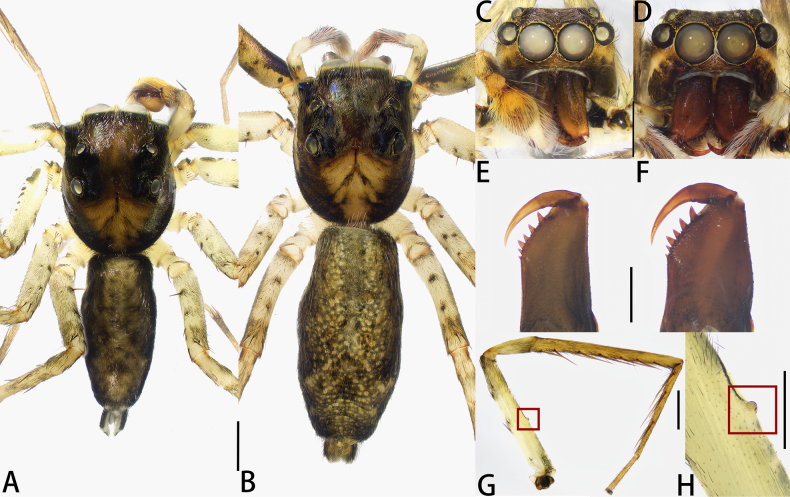
*Spartaeus yingdeensis* sp. nov. **A, C, E, G, H**. Male holotype; **B, D, F**. Female paratype (MHBU-ARA-00028589); **A, B**. Habitus, dorsal; **C, D**. Habitus, frontal; **E, F**. Left chelicera, posterior; **G, H**. Left leg I, prolateral. Scale bars: 1 mm (**A–D, G**); 0.5 mm (**E, F, H**).

#### Etymology.

The species epithet is derived from the type locality; it is treated as an adjective.

#### Diagnosis.

**Figure 3.**
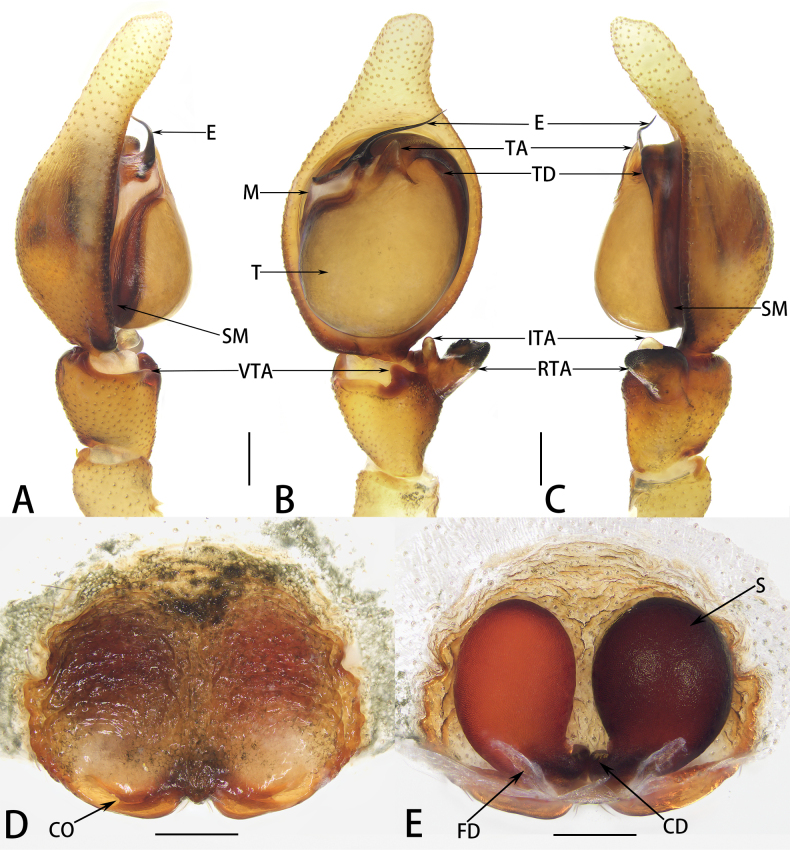
*Spartaeus yingdeensis* sp. nov. **A–C**. Palp of male holotype; **D, E**. Epigyne of female paratype (MHBU-ARA-00028589); **A**. Prolateral; **B, D**. Ventral; **C**. Retrolateral; **E**. Dorsal. Abbreviations: CD, copulatory duct; CO, copulatory opening; E, embolus; FD, fertilization duct; ITA, intermediate tibial apophysis; M, membrane of distal haematodocha; RTA, retrolateral tibial apophysis; S, spermatheca; SM, spermophor; T, tegulum; TA, tegular apophysis; TD, tegular depression; VTA, ventral tibial apophysis. Scale bars: 0.2 mm (**A–E**).

**Figure 4.**
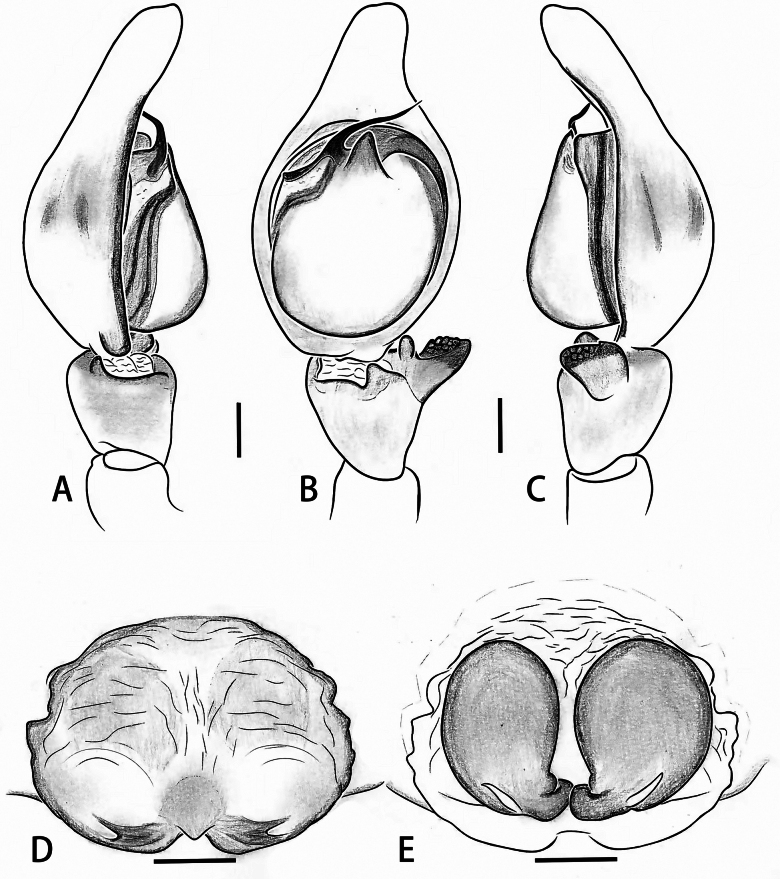
*Spartaeus yingdeensis* sp. nov. **A–C**. Palp of male holotype; **D, E**. Epigyne of female paratype (MHBU-ARA-00028589); **A**. Prolateral; **B, D**. Ventral; **C**. Retrolateral; **E**. Dorsal. Scale bars: 0.2 mm (**A–E**).

The new species resembles *S. pinniformis* Yang, Liu, Liu & Peng, 2017 in the shape of retrolateral tibial apophysis of male palp and the position of copulatory openings, but it can be distinguished by the combination of following characters: (1) the retrolateral tibial apophysis is broader with a bluntly rounded tip in retrolateral view (Figs 3C, 4C; whereas it is narrow and tapered distally in *S. pinniformis*; see Yang et al. 2017: fig. 4B); (2) the distal end of the intermediate tibial apophysis points prolaterally in ventral view (Figs 3B, 4B; whereas it points retrolaterally in *S. pinniformis*; see Yang et al. 2017: fig. 4A); (3) the embolus is long and slender, more than half the width of the bulb (Figs 3B, 3C, 4B, 4C; whereas it is short and stout, less than half the width of the bulb in *S. pinniformis*; see Yang et al. 2017: fig. 4A, B); (4) the tegular apophysis is elongate and narrow, with its distal end directing distally (Figs 3B, 4B; whereas it is broad and slightly serrated along the retrolateral margin, with its distal end directing retrolaterally in *S. pinniformis*; see Yang et al. 2017: fig. 4A); (5) the copulatory openings are separated by approximately one-third the epigynal width (Figs 3D, 4D; whereas separated by approximately one-fifth the epigynal width in *S. pinniformis*; see Yang et al. 2017: fig. 4D); (6) the spermathecae are subglobular (Figs 3E, 4E; whereas they are elongate-oval in *S. pinniformis*; see Yang et al. 2017: fig. 4E).

#### Description.

**Male**. Measurements of holotype: total length 6.66; carapace 2.89 long, 2.25 wide; abdomen 3.20 long, 1.47 wide; eye measurements: AME 0.69, ALE 0.38, PME 0.17, PLE 0.24; leg measurements: I 10.82 (3.17, 1.06, 3.10, 2.58, 0.91), II 7.35 (2.19, 0.95, 1.92, 1.55, 0.74), III 7.28 (2.00, 0.59, 1.85, 1.95, 0.89), IV 8.47 (2.45, 0.59, 2.04, 2.65, 0.74); leg formula: 1423. Carapace (Fig. 2A, C) light brown, anterior lateral margins and eye surroundings dark brown, centrally with inverted black Y-shaped mark; eye field covered with black setae; fovea dark, slit-like. Chelicerae (Fig. 2E) dark brown, with six promarginal and ten retromarginal teeth. Endites and labium yellowish brown, distally with black setae; endites slender; sternum marble-white. Legs (Fig. 2A, G, H) with long, thin spines; coxae and trochanters white; femora to tibiae laterally with light-grey stripes; femora with small, light yellow, spherical protrusion bearing translucent margin. Abdomen (Fig. 2A) yellow, margins with black stripes interspersed with white setae; dorsum dark yellow with black spots, white scaly patches, centrally covered with iridescent setae. Spinnerets (Fig. 2A) pale yellow, inner lateral sides white, exterior with dark patches, covered with black setae.

Palp (Figs 3A–C, 4A–C): ventral tibial apophysis thick, dark, triangular in ventral view; retrolateral tibial apophysis thick, stiff, hardly movable, appearing short and dark in ventral view, distally with small processes; intermediate tibial apophysis light brown, surface round and smooth; tegulum oval, surface smooth; tegular apophysis triangular, weakly sclerotized in ventral view; embolus whip-like, slender, base located in membrane formed by distal haematodocha, terminally sharp.

**Female**. Measurements of paratype (MHBU-ARA-00028589): total length 7.89; carapace 2.96 long, 2.32 wide; abdomen 4.24 long, 1.95 wide; eye measurements: AME 0.69, ALE 0.40, PME 0.24, PLE 0.33; leg measurements: I 9.92 (3.02, 1.00, 3.13, 1.92, 0.85), II 7.63 (2.30, 0.79, 2.04, 1.73, 0.77), III 7.57 (1.93, 0.72, 1.97, 2.20, 0.75), IV 9.65 (2.76, 0.98, 2.10, 2.99, 0.82); leg formula: 1423. Habitus similar to male but slightly larger. Carapace (Fig. 2B, D) medially paler, with black band extending from ALE to posterior margin; centrally with inverted black Y-shaped mark. Chelicerae (Fig. 2F) brown, with six promarginal and ten retromarginal teeth. Endites and labium yellowish brown to dark brown, distally with black setae; endites slender. Abdomen (Fig. 2B) lighter than in male; lateral dorsal black stripes discontinuous, forming intermittent small black bands. Spinnerets (Fig. 2B) dark yellow, darker than in male; inner lateral sides white, exterior with dark patches, covered with black setae.

Epigyne (Figs 3D, 3E, 4D, 4E): epigynal plate oval, slightly raised, mound-like; copulatory openings small, slit-shaped in ventral view, posteriorly located, facing laterally; copulatory ducts dark and thick, extending transversely at origin, then curved and finally connected to spermathecae; spermathecae reddish brown, large, pear-shaped; accessory glands not visible; fertilization ducts short, connected to spermathecae posteriorly, near copulatory ducts.

#### Distribution.

China (Guangdong).

### Spartaeus rotiscutus sp. nov.

Taxon classificationAnimaliaAraneaeSalticidae

1C644C4B-604E-552C-8B73-4FB1EF16B3D3

https://zoobank.org/09BD5D72-0B85-4FB2-A01C-EEF0F968FA36

Figs 5, 6, 7

#### Chinese common name.

圆盾散蛛.

#### Type material.

***Holotype***: China • ♂ (MHBU-ARA-00023867), Yunnan Province, Lincang City, Shuangjiang County, Ganlan Bay, 24.9616°N, 99.8584°E, 1421 m elev., 16.VI.2022, L. Zhang, W. Wang, M. Xu & Z. Yang leg. ***Paratypes***: China • 1♀ (MHBU-ARA-00028594), 1♀ (MHBU-ARA-00028595), same data as holotype • 1♀ (MHBU-ARA-00024334), 2♀ (MHBU-ARA-00023916), Shuangjiang County, Mengmeng Town, 23.4258°N, 99.8215°E, 1206 m elev., 14.VI.2022, L. Zhang, W. Wang, M. Xu & Z. Yang leg. • 1♀ (MHBU-ARA-00024051), Shuangjiang County, Mengmeng Town, Banqiao, 23.4258°N, 99.8215°E, 1206 m elev., 14.VI.2022, L. Zhang, W. Wang, M. Xu & Z. Yang leg.

#### Etymology.

The species epithet is a compound adjective in Latin (*rotundus* + *scutum*), meaning rounded shield. It refers to the tegulum and spermathecae, which resemble round shields.

#### Diagnosis.

The new species resembles *S. thailandicus* Wanless, 1984 in possessing a large, curved retrolateral tibial apophysis and the position of copulatory openings, but it can be distinguished by the combination of following characters: (1) the retrolateral tibial apophysis is gently curved with the tip directing laterally (Figs 6C, 7C; whereas it is strongly curved with the tip directing ventrally in *S. thailandicus*; see Wanless 1987: fig. 4F, I); (2) the intermediate tibial apophysis is distally thickened and bluntly rounded at the tip in ventral view (Figs 6B, 7B; whereas it is approximately uniform in thickness throughout in *S. thailandicus*; see Wanless 1987: fig. 4G); (3) the tip of ventral tibial apophysis is bluntly rounded (Figs 6B, 7B; whereas it is tapering to a papillate apex in *S. thailandicus*; see Wanless 1987: fig. 4G); (4) the membrane of distal haematodocha is short and broad, suboval (Figs 6B, 7B; whereas it is large and subtriangular in *S. thailandicus*; see Wanless 1987: fig. 4G); (5) the tegular apophysis is subtriangular (Figs 6B, 7B; whereas it is subrectangular in *S. thailandicus*; see Wanless 1987: fig. 4G); (6) the spermathecae are approximately 1.5 times as long as wide (Figs 6E, 7E; whereas they are approximately twice as long as wide in *S. thailandicus*; see Logunov and Azarkina 2008: figs 87, 90).

**Figure 5.**
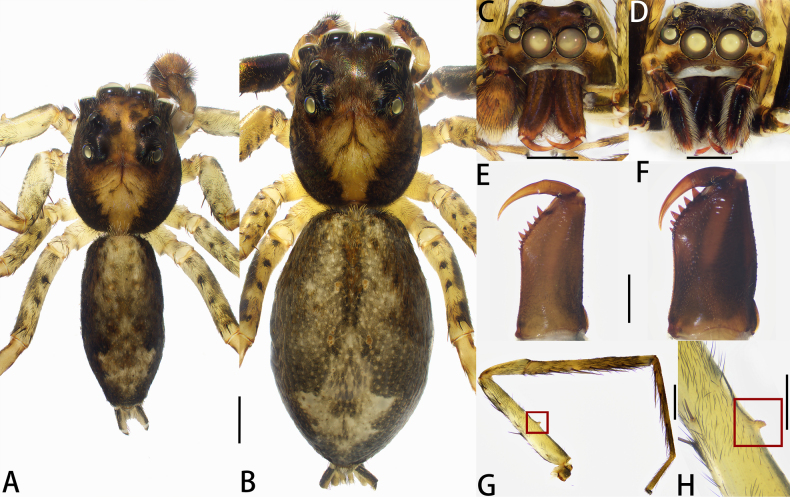
*Spartaeus rotiscutus* sp. nov. **A, C, E, G, H**. Male holotype; **B, D, F**. Female paratype (MHBU-ARA-00028594); **A, B**. Habitus, dorsal; **C, D**. Habitus, frontal; **E, F**. Left chelicera, posterior; **G, H**. Left leg I, prolateral. Scale bars: 1 mm (**A–D, G**); 0.5 mm (**E, F, H**).

**Figure 6.**
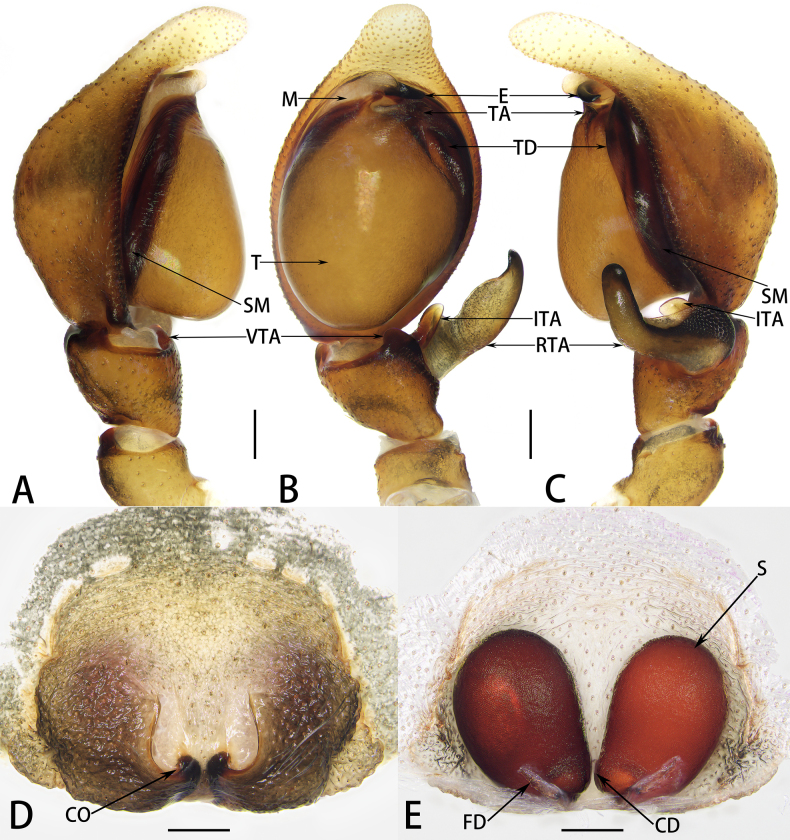
*Spartaeus rotiscutus* sp. nov. **A–C**. Palp of male holotype; **D, E**. Epigyne of female paratype (MHBU-ARA-00028594); **A**. Prolateral; **B, D**. Ventral; **C**. Retrolateral; **E**. Dorsal. Abbreviations: CD, copulatory duct; CO, copulatory opening; E, embolus; FD, fertilization duct; ITA, intermediate tibial apophysis; M, membrane of distal haematodocha; RTA, retrolateral tibial apophysis; S, spermatheca; SM, spermophor; T, tegulum; TA, tegular apophysis; TD, tegular depression; VTA, ventral tibial apophysis. Scale bars: 0.2 mm (**A–E**).

**Figure 7.**
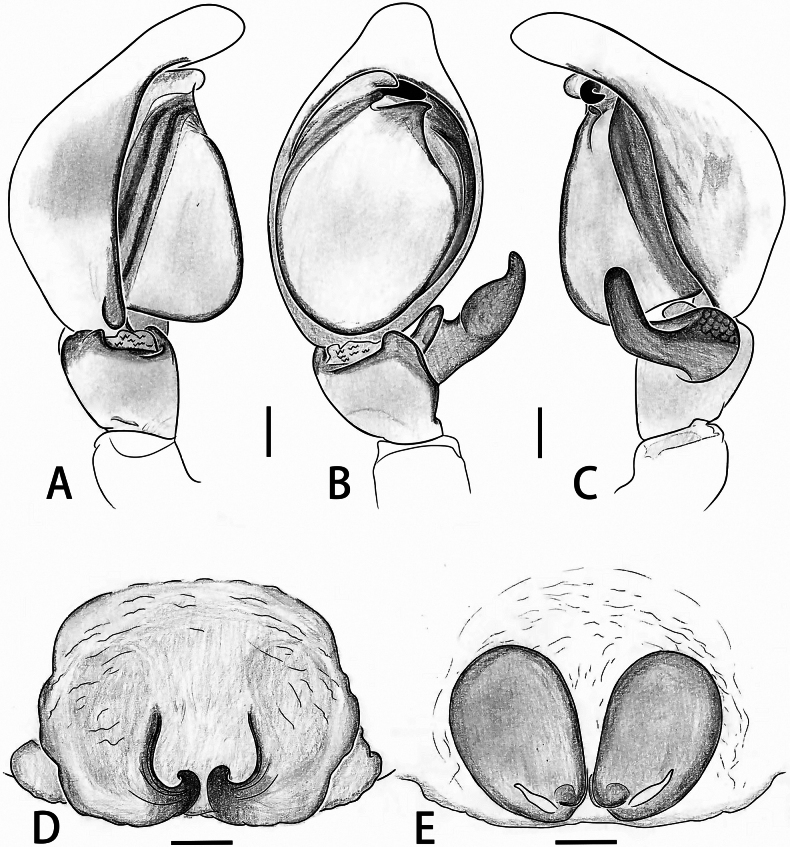
*Spartaeus rotiscutus* sp. nov. **A–C**. Palp of male holotype; **D, E**. Epigyne of female paratype (MHBU-ARA-00028594); **A**. Prolateral; **B, D**. Ventral; **C**. Retrolateral; **E**. Dorsal. Scale bars: 0.2 mm (**A–E**).

#### Description.

**Male**. Measurements of holotype: total length 6.93; carapace 2.79 long, 2.36 wide; abdomen 3.61 long, 1.78 wide; eye measurements: AME 0.60, ALE 0.31, PME 0.18, PLE 0.28; leg measurements: I 11.88 (3.23, 1.18, 3.55, 2.79, 1.13), II 7.52 (2.29, 0.94, 1.91, 1.68, 0.70), III 7.01 (1.86, 0.63, 1.51, 2.17, 0.84), IV 10.37 (2.75, 0.97, 2.70, 3.03, 0.92); leg formula: 1423. Carapace (Fig. 5A, C) yellow, margins and eye surroundings with dark-brown spots; posterolateral margins dark; eye field and eye margins with white and light-yellow setae; fovea dark, slit-like. Chelicerae (Fig. 5E) light brown; with seven promarginal and seven retromarginal teeth. Endites and labium yellowish orange, distally with black setae; endites slender. Legs (Fig. 5A, G, H) with long, thin spines; femora distally with small light-yellow, spot-like protrusion; coxae and trochanters greyish white; femora to tibiae anteriorly and posteriorly with distinct dark-grey spots. Abdomen (Fig. 5A) yellow, elongate-oval, scattered with black setae and dark-yellow spots; medially with grey scales; dorsum laterally with irregular, dark-brown mottling, forming indistinct longitudinal bands; venter pale yellow, uniformly coloured, covered with short, fine setae. Spinnerets (Fig. 5A) yellow, margins dark grey, inner lateral sides greyish white, with black setae.

Palp (Figs 6A–C, 7A–C): ventral tibial apophysis thick, hill-like in ventral view; retrolateral tibial apophysis soft, distally with dark blotches on anterior margin; intermediate tibial apophysis light brown, surface round, tip slightly membranous; tegular apophysis subtriangular in ventral view; embolus short and thick, rounded, and sheep-horn-like in retrolateral view; base hidden behind membrane formed by distal haematodocha.

**Female**. Measurements of paratype (MHBU-ARA-00028594): total length 9.36; carapace 3.45 long, 2.72 wide; abdomen 5.54 long, 3.45 wide; eye measurements: AME 0.74, ALE 0.36, PME 0.22, PLE 0.36; leg measurements: I 10.69 (3.19, 1.08, 3.36, 2.02, 1.04), II 8.26 (2.59, 0.82, 2.23, 1.79, 0.83), III 7.74 (1.97, 0.59, 1.98, 2.26, 0.94), IV 11.32 (3.19, 0.97, 3.05, 3.14, 0.97); leg formula: 4123. Carapace (Fig. 5B, D) medially light yellow, margins dark brown; centrally with black, inverted Y-shaped mark. Eye field light yellow to brown; eye margins with light-yellow parallel stripes, covered with yellow setae. Chelicerae (Fig. 5F) dark brown, anteromedially with brown patch; with six promarginal and eight retromarginal teeth. Endites and labium dark brown, distally with black setae; endites slender. Legs (Fig. 5B) yellowish brown, with long, thin, black spines; coxae and trochanters light yellow; femora to tibiae anteriorly and posteriorly with dark-grey stripes and patches. Abdomen (Fig. 5B) khaki, anteriorly interspersed with dark marginal stripes and black spots; dorsum with tawny patches and black setae. Spinnerets (Fig. 5B) yellow; inner lateral sides white, exterior dark grey, covered with black setae.

Epigyne (Figs 6D, 6E, 7D, 7E): epigynal plate oval, raised, mound-like, posteriorly with slightly hooked ridge; copulatory openings small, slit-shaped in ventral view, located behind hooked ridge; copulatory ducts dark, extending dorsally; spermathecae dark red to dark brown, large, bean-shaped; accessory glands not visible; fertilization ducts short, located on posterior margins of spermathecae, directed anterolaterally.

#### Distribution.

China (Yunnan).

## Supplementary Material

XML Treatment for Spartaeus

XML Treatment for Spartaeus yingdeensis

XML Treatment for Spartaeus rotiscutus

## Additional information

### Artificial Intelligence (AI) use

The authors accept full responsibility for the content of the manuscript, including the disclosure of any use of AI.

Regarding the use of AI in the preparation of this manuscript, the authors declare the following: GPT.

**Used for**: Language, style and writing.

GPT was only used for checking and correcting grammar errors in the manuscript.

### Author ORCIDs

Z. Luo https://orcid.org/0009-0003-1069-1087

Y. Ni https://orcid.org/0009-0003-3239-3697

J. Zhang https://orcid.org/0000-0003-2179-3954

### Data availability

All of the data that support the findings of this study are available in the main text.
